# Trans-tarsal stair-step versus transconjunctival approach in orbito-zygomaticomaxillary fractures: a prospective randomized controlled clinical trial

**DOI:** 10.1186/s12903-026-08842-1

**Published:** 2026-06-17

**Authors:** David Emad Sobhy, Noha Y. Dessoky, Amany M. Alryess, Marwa G. Noureldin

**Affiliations:** Oral and Maxillofacial Surgery Department, Faculty of Dentistry, Alexandria University, Champlion st, Azrite, Alexandria, Egypt

**Keywords:** Orbito-zygomaticomaxillary fracture, Transconjunctival Incision, Lower eyelid, Lateral canthotomy, Tarsal plate

## Abstract

**Background:**

Various techniques have been proposed for treating orbito-zygomaticomaxillary fractures, each with distinct benefits and drawbacks.

**Aim of this study:**

To compare Trans-tarsal Stair-Step Lateral Extension of the Transconjunctival approach with transconjunctival approach combined with lateral eyebrow incision in the management of orbital and zygomaticomaxillary complex fractures with respect to accessibility to fracture site and exposure duration as primary aims.

**Materials and methods:**

Twenty patients, aged between 18 and 60 years, who has orbito-zygomaticomaxillary complex fractures were randomly assigned to two equal groups: Group A underwent the trans-tarsal stair-step lateral extension of the transconjunctival approach and Group B received the conventional transconjunctival approach combined with lateral eyebrow incision. Intraoperative parameters which were accessibility to fracture site, exposure duration and total operative time and postoperative parameters which were postoperative edema, postoperative scar and esthetic appearance, postoperative pain and infraorbital sensory nerve function objectively, all were assessed.

**Results:**

Group A achieved same surgical access to the fracture site as Group B. Group A and group B had same exposure time. Group A had less total operative time than Group B. Group A consistently showed the less postoperative edema at first 48 h, one week, and four weeks. Non noticeable scarring was reported only in Group A patients while group B had a noticeable scar at the lateral eyebrow incision. Within the first 24 h, there was significant difference in pain levels that was noted between the two groups. Group A had faster recovery in sensory nerve function than group B.

**Conclusion:**

The trans-tarsal stair-step lateral extension of the transconjunctival approach demonstrated favorable postoperative outcomes regarding pain, edema, and sensory recovery compared to the conventional technique with lateral eyebrow incision with same exposure area and no increase in exposure time and less total operative time.

**Trial registration:**

The trial has been retrospectively registered on clinicaltrials.gov (ID: NCT07044258) on 19-6-2025.

**Supplementary Information:**

The online version contains supplementary material available at 10.1186/s12903-026-08842-1.

## Introduction

The orbito-zygomaticomaxillary complex (OMC) is a four-pointed structure that articulates with the maxilla, frontal, temporal, and sphenoid bones [1]. It is frequently affected in maxillofacial trauma, accounting for approximately 23% to 42% of such fractures, as reported in previous studies [2, 3]. As a key structural support of the face, the integrity of the OMC is essential for both functional and aesthetic aspects of the facial skeleton. Achieving proper anatomical repositioning of a fractured OMC requires sufficient exposure of the fracture site [4].

Inadequate reduction of the OMC can result in facial asymmetry, infraorbital nerve sensory disturbances, impaired ocular function, and/or limited mandibular movement [1]. Over 70% of OMC fractures are treated surgically using open reduction and internal fixation, with access to the inferior orbital rim achieved via either a transcutaneous or transconjunctival approach. Transcutaneous methods such as infraorbital, subtarsal, and subciliary incisions offer excellent exposure of the fracture site, but are associated with visible scar formation [5, 6]. The subciliary incision is placed approximately 2 mm below the lash line, whereas the subtarsal approach is made within a natural skin crease, typically 5 to 7 mm beneath the ciliary margin [7].

The transconjunctival approach is favored for its ability to avoid visible scarring in exposed facial areas. A key advantage of this technique is that it allows clear visualization of the orbital floor and inferior orbital rim while preserving the integrity of the lacrimal drainage system [8]. This technique is performed via the conjunctiva below the tarsal plate and followed by either preseptal or retroseptal dissection to the orbital rim, it has also been shown to be a convenient and time-efficient approach [1]. However, certain limitations have been noted regarding access for fracture reduction and plate fixation. Moreover, this approach demands technical expertise, adequate eye protection, and careful tissue retraction to minimize the risk of complications [9].

With the transconjunctival approach, a frequently reported drawback of this approach is its restricted intraoperative visibility, particularly when broader exposure of the orbital rim is required for fracture fixation or when access to the lateral buttress is necessary for reducing Le Fort or orbito-zygomatic fractures [10, 11]. Traditionally, this limitation has been addressed by performing a lateral extension of the incision through lateral canthotomy and cantholysis [12].

In 1994, Chalain et al. introduced a modification to the transconjunctival approach involving a lateral paracanthal incision, in which only the inferior limb of the lateral canthus is transected [13, 14]. More recently, this technique was revisited in East Asia by Kim et al. and Song et al., who modified it by making an incision 2 to 3 mm medial to the lateral canthus, through the tarsal plate, thereby avoiding disruption of the lateral canthus itself [15–17].

To the authors’ knowledge, there are no published studies that have specifically compared exposure duration and surgical accessibility between the trans-tarsal stair-step lateral extension of the transconjunctival approach (Trans-tarsal) and the conventional transconjunctival approach combined with lateral eyebrow incision in the management of orbito-zygomaticomaxillary complex fractures.

Therefore, this study compared the trans-tarsal stair-step lateral extension of the transconjunctival approach to the conventional transconjunctival approach combined with lateral eyebrow incision with respect to accessibility to fracture site and exposure duration as primary aims. Exposure duration was assessed quantitatively. Also, to compare the clinical outcomes of the two surgical approaches in terms of postoperative functional and esthetic outcomes. We hypothesized that there would be no significant difference between the two approaches in terms of surgical accessibility, exposure time and the postoperative functional and esthetic outcomes.

## Patients and methods

### Study design and registration

This study is prospective randomized controlled clinical trial that was conducted based on the CONSORT Guidelines (http://www.consort-statement.org*).* The study was registered retrospectively at ClinicalTrials.gov (NCT07044258) on 19 June 2025. Also, the study was ethically approved by the Research Ethics Committee of Alexandria University Faculty of Dentistry (IRB NO. 001056 – IORG 0008839).

### Study setting and location

The participants were selected from the Emergency Ward of Alexandria university teaching hospital from October 2024 to February 2025 and operated under the authority of the oral and maxillofacial surgery department, faculty of dentistry, Alexandria University. Patients were informed about the procedure’s details and each patient signed an informed consent.

### Study sample

The sample size was calculated based on exposure time as it is one of our primary outcomes. Also, as it represents an objective and reproducible intraoperative parameter. This variable was selected due to its clinical relevance, as reported in previous studies [1, 9, 18], and its suitability for reliable power calculation compared to more subjective outcomes.

The sample size was calculated assuming a 95% confidence level and 80% study power, using operative time (from incision to fracture exposure) as the primary outcome due to its objectivity and reproducibility. Based on previously reported data based on previously published from a comparable surgical approach with a similar exposure area which was Transconjunctival with Y-Shaped extension (14.6 ± 2.31 min vs. 20.0 ± 3.41 min), and using the highest standard deviation to ensure adequate power, the minimum required sample size was estimated to be 8 patients per group. This was increased to 10 per group to compensate for potential dropouts, resulting in a total of 20 patients. The calculation was performed using Rosner’s method and verified using G*Power software (version 3.1.9.7) [19].

### Eligibility criteria for patient selection

#### Inclusion criteria

Preferable age of the selected patients was between 18 and 60 years with no gender predilections. In addition, only patients with an intact globe, stable ocular condition, and preserved visual potential were included.

#### Exclusion criteria

Patients with existing lacerations in the inferior or lateral periorbital regions, active infection at the fracture line, or acute or chronic conjunctival diseases were excluded. Also, patients with severely comminuted multi-fragmented zygomatic complex fractures with marked disruption of the orbital and zygomatic anatomy were excluded from the study. Additionally, patients with preoperative eyelid complications were excluded from the study.

### Randomization

Patients who met the inclusion criteria were allocated in a 1:1 ratio using an on-site computer software system with concealed allocation through sequentially numbered, opaque, sealed envelopes (SNOSE). Randomization was conducted with 2 & 4 random block sizes (http://www.randomizer.org/).

1. Group A: Trans-tarsal stair-step lateral extension of the transconjunctival approach.
2. Group B: Transconjunctival approach combined with lateral eyebrow incision.

### Grouping of the patients

- Group A: (*n* = 10) Ten patients were treated with trans-tarsal approach
- Group B: (*n* = 10) Ten patients were treated with transconjunctival approach combined with lateral eyebrow incision.

The control group was assigned to the transconjunctival approach combined with a lateral eyebrow incision. This approach provides adequate exposure while preserving the integrity and contour of the lateral canthal region. In contrast, techniques involving lateral canthotomy require disruption of the lateral canthal angle, and its reconstruction may not consistently restore the original contour as it has non-reliable and non- consistent landmark, with reported complications such as canthal rounding, eyelid malposition and ectropion. Therefore, this approach was selected to maintain canthal anatomy and contour. Also, to reduce confounding variables associated with canthotomy [16, 17, 20–22].

## Methods

### Preoperative assessment

Complete personal information, past medical and dental histories, and the chief complaint were documented, with particular attention to the cause, timing, date, location, and mechanism of the assault. Clinical examination of all patients included systematic extraoral and intraoral assessment performed through visual inspection and palpation. Computed tomography imaging was routinely performed preoperatively for all enrolled patients.

### Preoperative patient preparation

Required preoperative laboratory tests were performed for surgical clearance, and patients were advised to maintain an 8-hour fasting period before surgery.

Cefotaxime (Cefotax: each vial contains cefotaxime (as sodium salt) 1 gm, manufactured by E.I.P.I.C.O.)1gm/12 intravenously were given preoperatively as prophylaxis to prevent postoperative infection.

### Operative procedure

All procedures were performed to the whole patients by the same surgeon to ensure consistency (D.S). The patients were operated under general anesthesia and the surgical field was disinfected with povidone iodine solution, then draped with sterile towels showing the site of surgery only.

#### Group A

**Fig. 1:**
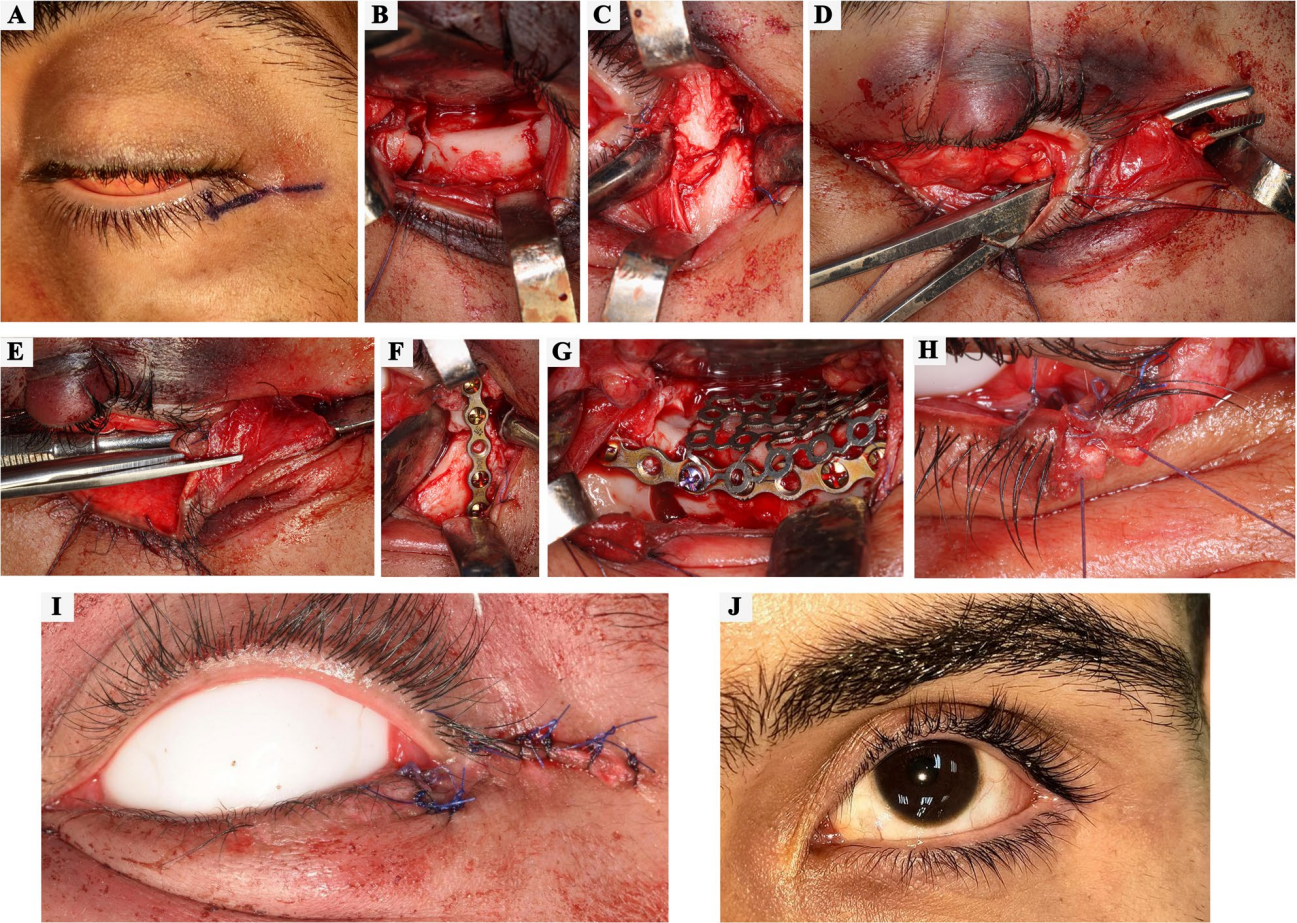
Surgical steps of the trans-tarsal stair-step lateral extension of the transconjunctival approach (Group A): (**A**) Landmark is drawn. **B** Exposure field including infraorbital rim and orbital floor fractures through transconjunctival alone without the extension. **C** Exposure of the lateral orbital rim fracture through the extension alone. **D** The through and through dissection between the transconjunctival approach and the lateral extension by curved mosquito. **E** Doing the trans-tarsal stair-step incision by cutting the eyelid margin to connect the whole incision together. **F** Reduction and fixation of the lateral orbital rim. **G** Reduction and fixation of the infraorbital rim and orbital floor. **H** Suturing of the inferior border of the tarsus plate. **I** The skin sutures. **J** Postoperative follow up

The surgery was performed as previously described [17, 21, 23]. A corneal shield was placed on the operative eye at the beginning of surgery to ensure corneal protection during incision. Starting 5 mm medial to the lateral canthus, a perpendicular mark was placed relative to the lower eyelid. From this point, a line was drawn beneath the tarsal plate and extended laterally along the crow’s feet crease, reaching a line tangent to the lateral margin of the eyebrow. Local anesthetic with vasoconstrictor was injected subcutaneously along the marked line at the zygomatic area crossing the infraorbital rim. Using forceps and traction sutures 4–5 mm below the lid margin, the lower eyelid was everted. A post-septal conjunctival incision was made without extending medially beyond the lacrimal punctum to keep the healing scar away from the tarsal plate and margin. Using a curved hemostat, blunt dissection was performed along the infraorbital rim. A 5–0 prolene suture was applied at the palpebral margin to safeguard the canthal apparatus. Through a transverse transcutaneous incision 2 mm lateral to the lateral canthus along a predetermined rhytid over the lateral orbital rim, the muscle was vertically dissected in alignment with its fibers. Following exposure of the fracture, dissection was carried down to the lateral orbital rim and extended subperiosteally medially to integrate with the transconjunctival approach plane. Perpendicular to the eyelid margin, a trans-tarsal incision approximately 5 mm medial to the lateral canthus was created to connect with the transconjunctival incision, completing the “stair-step”. A gingivolabial approach was done intraorally to expose the zygomaticomaxillary fracture. Reduction and fixation of fractures in a regular fashion was accomplished using mini or micro plates. Using 6/0 vicryl suture, Place a vertical mattress suture across the cut tarsal edges. The bites should be partial thickness, passing through the anterior two-thirds of the tarsus to avoid penetrating the conjunctiva. Then the eyelid margin is aligned by placing a vertical mattress suture through the Meibomian gland orifices (gray line) on both sides of the tear. This ensures the eyelashes are perfectly aligned. Another suture was added at the margin to provide eversion. The conjunctiva was closed with wide-bite continuous 6/0 Vicryl sutures to increase spacing between consecutive continuous sutures to limit scarring and lower the risk of entropion. The inferior tarsal plate was sutured with simple interrupted 6/0 Vicryl, followed by closure of the palpebral orbicularis oculi with inverted interrupted 6/0 Vicryl, and the lateral muscle extension with inverted 5/0 Vicryl. Skin closure was completed using simple interrupted 6/0 Prolene sutures, removed after 7–10 days. The intraoral wound was closed via 3/0 vicryl sutures. The eye was irrigated with saline, and a Frost suture was applied for 1 week Fig. 1.

#### Group B

The surgery was performed as previously described [24, 25]. The conventional transconjunctival approach was made in the same manner as group A with no further extensions or modifications to access the infraorbital rim and orbital floor. Lateral eyebrow incision was done to access the lateral orbital rim. A gingivolabial approach was done intraorally to expose the zygomaticomaxillary fracture. Reduction and fixation were done same as group A. The conjunctiva was sutured as was explained in group A. Layered closure of the lateral eyebrow incision was performed, starting with the periosteum, then the muscles with 4/0 Vicryl, and finally the skin using subcuticular 5/0 Prolene sutures, the intraoral wound was closed via 3/0 vicryl sutures, removed 7–10 days postoperatively.

### Post-operative phase

Early postoperative care All patients were advised on application of ice pack extra-orally immediately postoperatively for 12 h. Also, a high protein, soft, high calorie diet was instructed for all patients postoperatively Postoperative medication was intravenous cefotaxime 1 gm/12 h on the first day then amoxicillin + clavulanate (Augmentin: amoxicillin 875 mg + clavulanic acid 125 mg: GlaxoSmith kline, UK) 1 gm twice daily for 5 days, Metronidazole (Flagyl: metronidazole 500 mg by GlaxoSmithkline, UK) 500 mg every 8 h for 5 days, Alpha-chemo-trypsin (Alpha-chemo-trypsin: Leur quin France, packed by Amoun pharmaceutical CO.S.A.E-Egypt) ampules once daily for 5 days, Diclofenace potassium (Cataflam: diclofenacpotassium 50 mg: Novartis-Switzerland) 50 mg for 5 days. Patients were advised to use antiseptic chlorhexidine (Hexitol: chlorhexine125mg/100 ml, concentration 0.125%: Arabic drug company, ADCO) mouth wash in situations where additional vestibular maxillary intraoral incision was performed. Eye lubricant drops for refreshment of the eye (Solofresh sodium hyaluronate 0.2%: Orchidia pharmaceutical industry). Tobramycin and dexamethasone eye drops (TubraDex eye drops: Novartis). Tobramycin and dexamethasone cream around the eye (Tubradex cream: Alcon).

### Parameters for evaluation

However, the current study had several limitations, including the inability to implement blinding for either patients or operators due to the obvious nature of the incision, as well as the relatively short follow-up period.

#### Intraoperative evaluation

Accessibility to fracture site was the assessment of the incision was provided adequate exposure and accessibility to reduction and fixation of the fracture [21].

The time taken between performing the incision till exposure of the field was recorded using a stopwatch [26].

The time taken within the whole surgery was recorded using a stopwatch [26].

#### Postoperative clinical evaluation

Clinical evaluation was conducted by a blinded assessor not involved in the operative procedure nor group allocation (A.A). However, complete assessor blinding was not always feasible due to the presence of visible external skin incisions in some cases. Patients were evaluated inorder to assess the following parameters at 24 h, 48 h, 1 week, 3 weeks, 4 weeks and the Infra-Orbital Sensory nerve was up to 12 weeks:

- The postoperative edema was categorized subjectively into mild (just noticeable), mild to moderate (more obvious edema without occlusion of palpebral fissure), moderate to severe (edema partially occluding palpebral fissure), and severe (edema totally occluding palpebral fissure) [26, 27].
- Postoperative scarring was assessed as noticeable or unnoticeable at 6 weeks postoperatively and thus esthetic affection was evaluated [9].
- Pain was evaluated through a 10-point Numerical Rating Scale (NRS), (0 1 = none, 2–4 = mild, 5–7 = moderate,8–10 = severe) [28].
- Sensory nerve function was evaluated by objective assessment which was done by using a Pin prick test (nociceptive method). The specific sites include mid-way of the dimensions of the lower eye lid, middle of the lateral part of the nose, middle portion of the upper lip and middle of zygoma [27].

#### Radiographic evaluation

Radiographic evaluation was performed using immediate postoperative Computed Tomography (CT) scans as part of routine postoperative assessment to confirm the adequacy of fracture reduction and fixation.

### Statistical analysis

The statistical analysis of the data was performed using IBM SPSS software version 20.0 (Armonk, NY: IBM Corp, released 2011). Categorical data were summarized as numbers and percentages. For continuous data, normality was assessed using the Shapiro-Wilk test. Continuous variables were assessed for normality using the Shapiro-Wilk test. Exposure time was normally distributed and was analyzed using the Student t-test, whereas pain scores were not normally distributed and were analyzed using the Mann–Whitney test. Quantitative data were described using range (minimum and maximum), mean, standard deviation, median and interquartile range (IQR). Significance of the results obtained was judged at the 5% level. Due to the small sample size, the results of normality testing should be interpreted with caution, as non-significant findings do not necessarily confirm a normal distribution.

The tests used were Chi-square test was used for categorical variables, to compare between different groups. Fisher’s Exact or Monte Carlo correction were used for Correction for chi-square when more than 20% of the cells have expected counts less than 5. Student T-test was used for normally distributed quantitative variables, to compare between two studied groups. Mann Whitney test was used for non-normally distributed quantitative variables, to compare between two studied groups.

## Results

A total of twenty patients, aged 18 to 60 years, were included in this study. They were recruited from the Emergency Ward of Alexandria University Teaching Hospital and underwent surgical management under the supervision of the Oral and Maxillofacial Surgery Department, Faculty of Dentistry, Alexandria University.

### Clinical evaluation

#### Accessibility to fracture site Table 1

In terms of fracture-site accessibility, all groups achieved easy access, and no statistically significant difference was observed between the two groups in term of accessibility to the fracture site.

**Table 1:** Description of the two studied groups according to accessibility to the fracture site intraoperative

	Study	Control
Patient 1	Infraorbital rim + lateral orbital rim + orbital floor	Infraorbital rim + lateral orbital rim + orbital floor
Patient 2	Infraorbital rim + lateral orbital rim + orbital floor + temporal process of zygomatic bone	Infraorbital rim + lateral orbital rim + orbital floor
Patient 3	Infraorbital rim + lateral orbital rim + orbital floor	Infraorbital rim + lateral orbital rim
Patient 4	Infraorbital rim + lateral orbital rim	Infraorbital rim + lateral orbital rim + orbital floor
Patient 5	Infraorbital rim + lateral orbital rim + orbital floor	Infraorbital rim + lateral orbital rim + orbital floor
Patient 6	Infraorbital rim + lateral orbital rim + orbital floor	Infraorbital rim + lateral orbital rim
Patient 7	Infraorbital rim + lateral orbital rim + orbital floor	Infraorbital rim + lateral orbital rim + orbital floor
Patient 8	Infraorbital rim + lateral orbital rim + orbital floor	Infraorbital rim + lateral orbital rim + orbital floor
Patient 9	Infraorbital rim + lateral orbital rim + orbital floor	Infraorbital rim + lateral orbital rim + orbital floor
Patient 10	Infraorbital rim + lateral orbital rim + orbital floor	Infraorbital rim + lateral orbital rim

#### Exposure duration Table 2

With respect to the duration from incision to surgical field exposure, no statistically significant difference was observed in terms of exposure time duration.

**Table 2:** Comparison between the two studied groups according to exposure duration from incision to reach the fracture

	Study(*n* = 10)		Control(*n* = 10)		Test of Sig.	*p*
	No.	%	No.	%		
Duration (min)						
Min. – Max.	14.0–20.0		14.0–20.0		t = 0.370	0.716
Mean ± SD.	16.40 ± 1.78		16.10 ± 1.85			
Median (IQR)	16.5 (15.0 − 17.0)		16.0 (15.0–17.0)			

*IQR *Inter quartile range, *SD *Standard deviation, *t *Student t-test, χ2 Chi square test, *FE *Fisher exact sheet

*p*: *p* value for comparing the two studied groups

*: Statistically significant at *p* ≤ 0.05

#### Total operative time Table 3

With respect to the duration of the surgery there was statistical significance between the 2 studied groups. When comparing the 2 groups, Group A had less operable time than group B.

**Table 3:** Comparison between the two studied groups according to total operative time

	Study (*n* = 10)	Control (*n* = 10)		T	p
Operative time (Min)					
Min. – Max.	170.0–235.0	250.0–283.0	t = 6.02		0.001^*^
Mean ± SD.	205.00 ± 20.82	264.90 ± 12.83			
Median (IQR)	207.5 (190–220)	262.0(253.0–277.0)			

*IQR *Inter quartile range, *SD *Standard deviation, *t *Student t-test

*p*: *p* value for comparing between the two studied groups

*: Statistically significant at *p* ≤ 0.05

#### Post-operative edema Fig. 2

The decrease in the edema value was recorded in both groups were recorded at 24 h, 48 h, 1 week, 2 weeks, 3 weeks, and 4 weeks postoperatively, and these values were statistically significant. When comparing the two groups, edema was significantly lower in Group A than in Group B during all of these postoperative intervals.

**Fig. 2:**
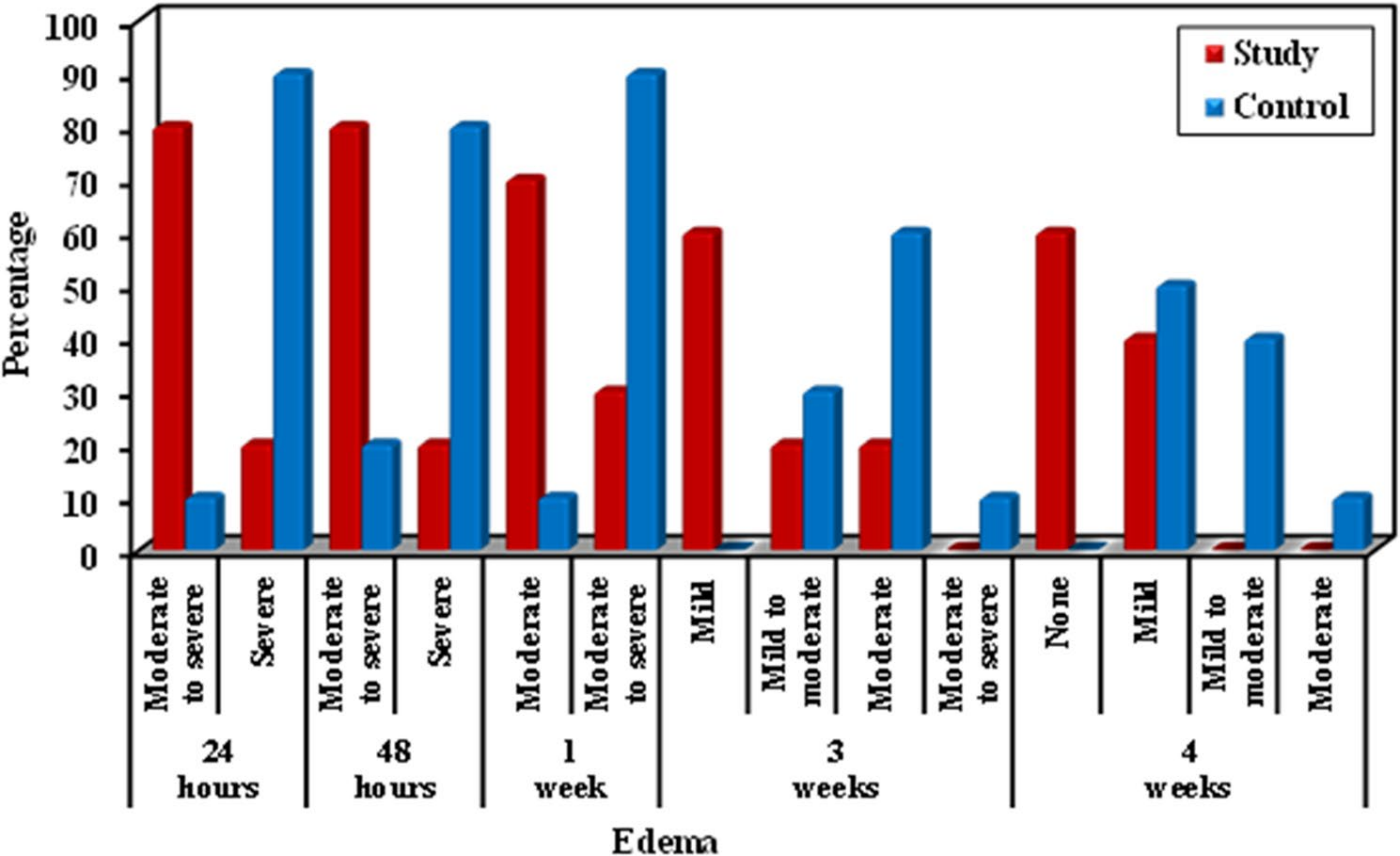
Comparison between the two studied groups according to postoperative edema

#### Postoperative scar and esthetic appearance Fig. 3

Regarding scarring, group A showed acceptable early postoperative scar appearance than group B.

**Fig. 3:**
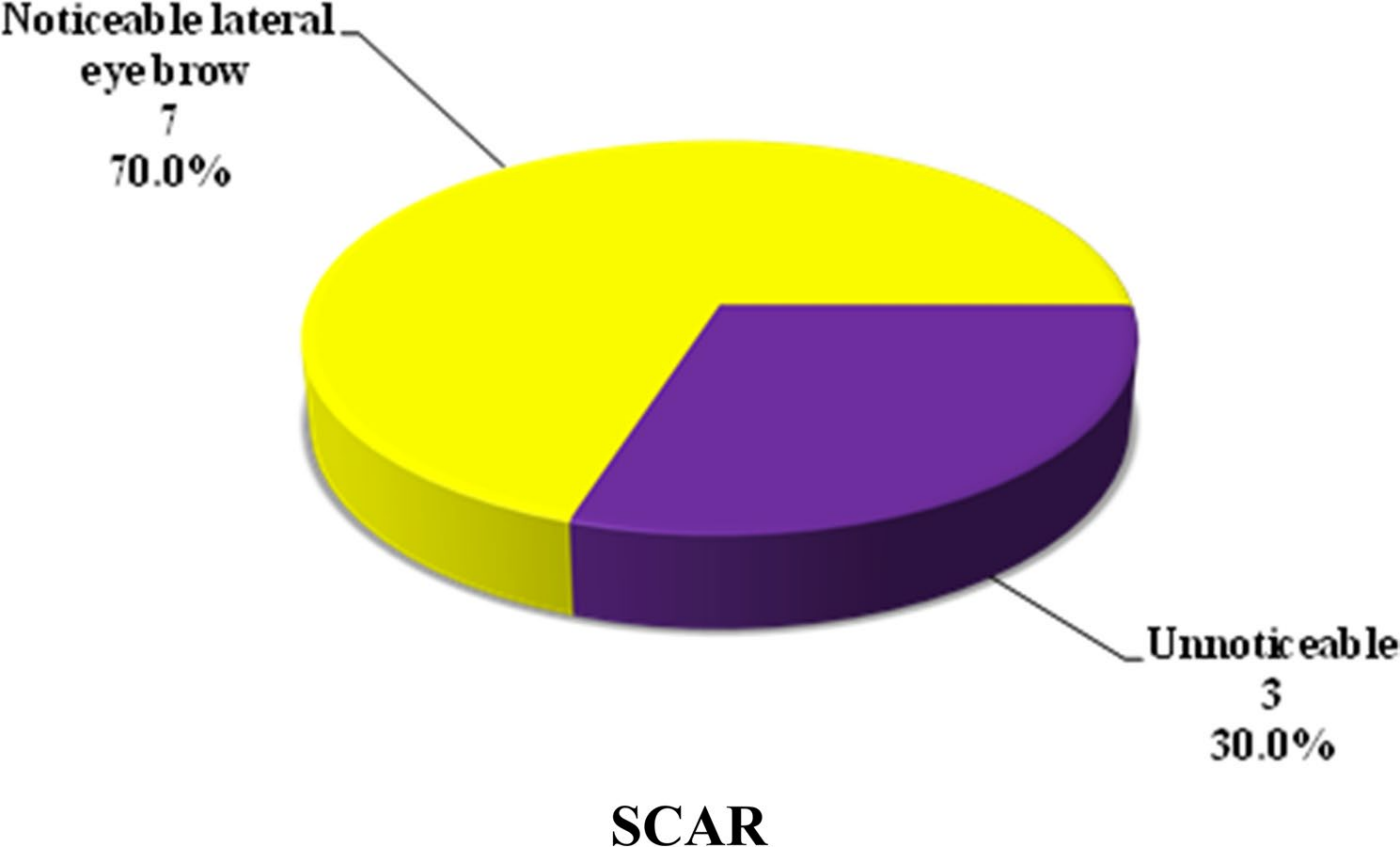
Distribution of studied sample according to postoperative scar and esthetic appearance in Control group (*n* = 10)

#### Post-operative pain Table 4

Pain levels during the first 24 h, 48 h, 1 week, 2 weeks, and 3 weeks showed a statistically significant difference between groups A and B. From the fourth week onward, no significant difference was detected between the two groups.

**Table 4:** Comparison between the two studied groups according to pain score

Pain score	Study (*n* = 10)	Control (*n* = 10)	U	*p*
24 h			4.000*	< 0.001^*^
Min – Max	4.0 – 6.0	6.0 – 9.0		
Mean ± SD	5.20 ± 0.79	7.30 ± 0.95		
Median (IQR)	5.0 (5.0 – 6.0)	7.0 (7.0 – 8.0)		
48 h			19.000^*^	0.019^*^
Min – Max	4.0–6.0	4.0–7.0		
Mean ± SD	4.80 ± 0.79	6.0 ± 1.05		
Median (IQR)	5.0 (4.0–5.0)	6.0 (5.0–7.0)		
1 week			11.000^*^	0.002^*^
Min – Max	1.0–4.0	3.0–5.0		
Mean ± SD	2.50 ± 0.85	4.0 ± 0.82		
Median (IQR)	2.50 (2.0–3.0)	4.0 (3.0–5.0)		
3 weeks			14.000^*^	0.005^*^
Min – Max	0.0–3.0	2.0–3.0		
Mean ± SD	1.10 ± 0.99	2.40 ± 0.52		
Median (IQR)	1.0 (0.0–2.0)	2.0 (2.0–3.0)		

*IQR* Inter quartile range, *SD *Standard deviation, *U *Mann Whitney test

*p*: *p* value for comparing between the two studied groups

*: Statistically significant at *p* ≤ 0.05

#### Infraorbital sensory nerve function objectively Fig. 4

With respect to sensory recovery objectively, a statistically significant difference was observed between the two groups at the 1st, 3rd, 4th, and up to the 6th week postoperatively. Group A demonstrated faster return of sensation compared to Group B. At 12 weeks, both groups showed non statistically significant difference.

**Fig. 4:**
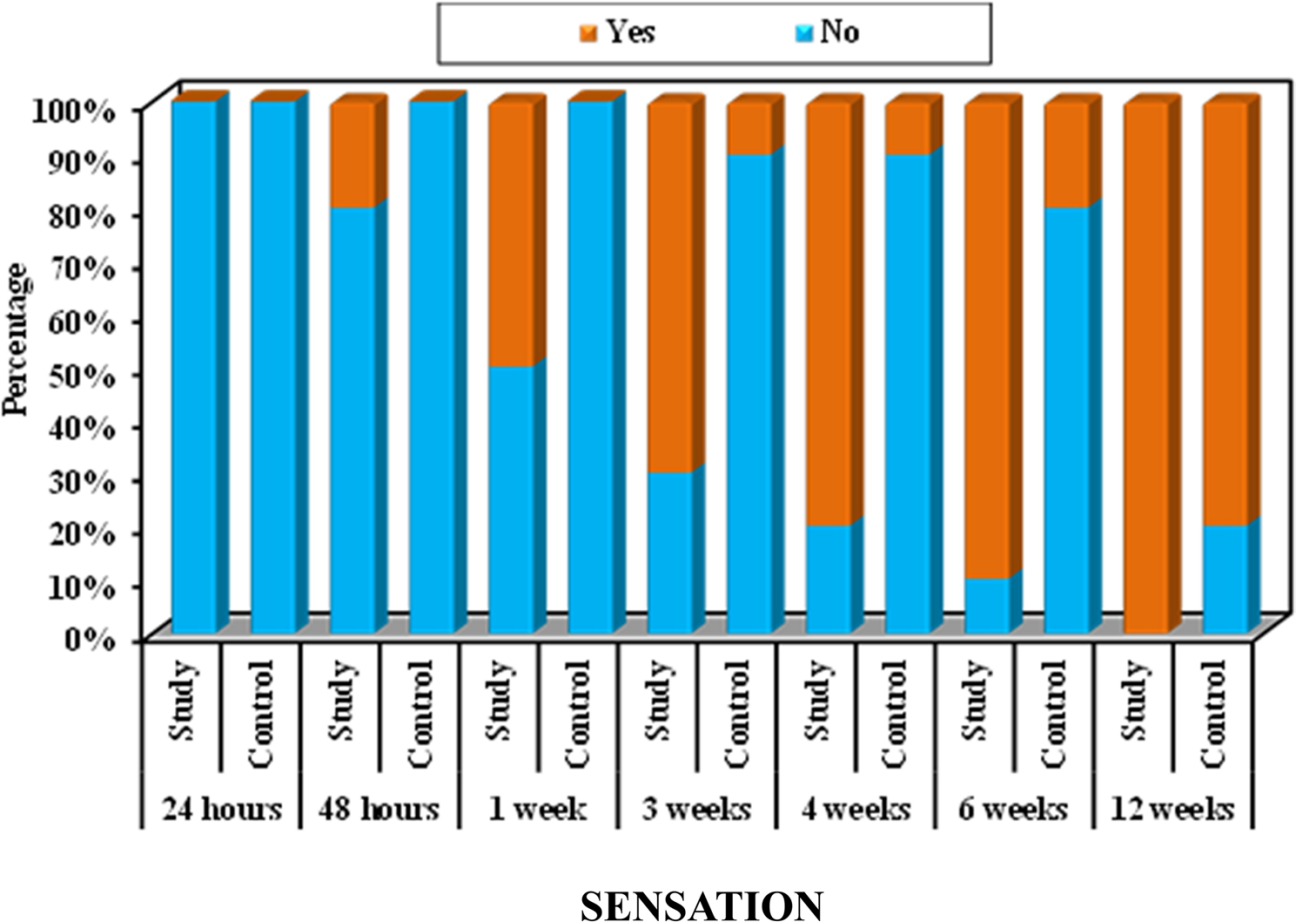
Comparison between the two studied groups according to infraorbital sensory nerve function by objective method

### Radiographic evaluation: (Supplementary Fig. S1)

Immediate postoperative CT scans were routinely performed to confirm the adequacy of fracture reduction and fixation. Representative image of postoperative 3-dimensional (3D) CT is provided in the Supplementary Material.

## Discussion

A variety of surgical incisions have been utilized in the management of OMC fractures to expose the orbital rim. These approaches have been extensively evaluated to identify the one offering optimal accessibility while minimizing postoperative complications. The aim of our study was to compare two surgical approaches: the trans-tarsal stair-step lateral extension of the transconjunctival approach and conventional transconjunctival approach combined with lateral eyebrow incision.

The sample size was calculated based on an expected difference in operative time; however, the observed difference between groups was smaller. This should be considered when interpreting the findings.

Regarding surgical accessibility, no statistically significant differences were observed between the two groups regarding surgical accessibility. However, these findings should be interpreted with caution in light of the relatively small sample size, which may limit the ability to detect smaller but clinically relevant differences. The 2 groups in our study provided comparable and straightforward access to the infraorbital rim, orbital floor, and lateral orbital rim. It’s worth noting that the cases reported as having exposure limited to the infraorbital and lateral orbital rims were not a result of inadequate surgical access. Rather, this finding was related to the presence of an intact orbital floor, which did not necessitate further exposure during fracture reduction and fixation. Although exposure could not be quantitatively measure, the Tran-tarsal extension consistently provided same similar qualitative exposure with just easier placement and adaptation of the titanium mesh compared to the conventional transconjunctival approach combined with lateral eyebrow incision.

This result aligns with the findings of Shannon R Garvey et al., who demonstrated in a study of 10 cases that the trans-tarsal stair-step lateral extension of the transconjunctival approach provides excellent visualization of the orbital floor without compromising the lacrimal drainage system or lateral canthal tendons. They further concluded that it represents the optimal surgical approach in terms of both functional and esthetic outcomes and reported no long-term complication [21]. El-Anwar et al. reported that the transconjunctival approach alone did not provide adequate exposure, necessitating a lateral canthotomy due to the limited surgical field [9]. The reported data of El-Anwar et al. concerns with our study. Both approaches provided comparable surgical exposure of the orbito-zygomaticomaxillary complex. The trans-tarsal stair-step extension allowed ease of access while maintaining minimal soft tissue disruption. We chose the transconjunctival approach combined with a lateral eyebrow incision for the control group to preserve the integrity of the lateral canthal region and the canthal apparatus of the orbit. This strategy avoids additional disruption that could occur with more invasive lateral canthotomy techniques, which have been associated with higher complication rates, including eyelid malposition [16, 17, 20–22, 29–31].

No statistically significant differences were observed regarding exposure time. However, these findings should be interpreted with caution in light of the relatively small sample size, which may limit the ability to detect smaller but clinically relevant differences. The study showed that operative time from incision to fracture exposure was comparable between the two groups where there was even considering the additional lateral eyebrow incision required in group B for lateral orbital rim access. Group A had the exposure duration (16.40 ± 1.78), while group B had the exposure duration (16.10 ± 1.85). A mean exposure duration of 21 min was recorded by Santosh and Giraddi for the transconjunctival approach, indicating a longer operative time [32]. The difference between Santosh and Giraddi and our study in exposure time still demonstrated a comparable time. While quantitative data on operative exposure time remain limited, the present study offers additional insight into this underexplored aspect of lateral extensions of the transconjunctival approach. But there was a clinical significance between the 2 groups within the total operative time as the mean time in group A was (205.00 ± 20.82) and group B (264.90 ± 12.83). Group B had much longer operative time which was related to the narrow field of the transconjunctival approach which needed more retraction to do proper reduction and fixation [9]. This also contributes to the increased postoperative edema in group B as the operation time is one of the factors affecting it [9]. Although exposure time was comparable between groups, the increased total operative time observed in Group B likely reflects the cumulative effect of more constrained surgical access, repeated intraoperative manipulation, and the additional time required for layered closure of the secondary incision.

Group A demonstrated the least postoperative edema at 24 h, one week, and four weeks, with statistically significant differences compared to group B. This finding can be attributed to the prolonged time required in group B for retraction, reduction, and fixation, as the conventional transconjunctival approach provides a relatively narrow field of exposure to the infraorbital rim and orbital floor resulting in excessive postoperative edema especially inferior to the orbit [33]. Although the lateral orbital rim in group B was easily accessed through the lateral eyebrow incision, the limited exposure of the infraorbital region necessitated more extensive retraction [33]. In contrast, group A benefited from a wide, continuous exposure that allowed clear visualization of the infraorbital rim, orbital floor, and lateral orbital rim without excessive tissue retraction, thereby reducing the operative time needed for reduction and fixation [21]. In the present study, postoperative periorbital edema was notably lower in the trans-tarsal approach group compared with conventional transconjunctival cases. This is likely due to reduced tissue retraction, as the stair-step incision provides a broader and continuous exposure. Previous studies have highlighted that excessive tissue retraction, rather than operative time alone, is a major contributor to periorbital edema in transconjunctival approaches [9]. It is important to emphasize that the operative time associated with reduction and fixation is distinct from exposure duration. Exposure duration reflects only the time required to access and visualize the fracture site and was comparable between both approaches. In contrast, the manipulation needed during reduction and fixation, particularly in narrower surgical fields, may be relatively longer. This increased operative handling can contribute to postoperative edema. Therefore, the observed edema reflects the operative manipulation rather than exposure time.

In our study, the trans-tarsal approach provided excellent access to both the inferior and lateral orbital rims in all 10 cases of group A, eliminating the need for an additional lateral eyebrow incision or lateral canthotomy preserving both the lateral canthal angle and the lateral canthal tendon. By this extension we were able to reach the whole lateral orbital rim without forced retraction to the tissues [21, 23]. while in group B we needed to do a lateral eye brow incision to reach the lateral orbital resulting in a noticeable scar [23]. This agrees with Kazuya Kashiyama et al. who reported pigmentation and scaring at the lateral eyebrow incision [34].

Our findings showed an encouraging early postoperative esthetic findings in Group A than Group B. The group of trans-tarsal approach had 0 cases of unnoticeable scar while the conventional transconjunctival approach had 7 cases of noticeable scar at the lateral eyebrow incision. The trans-tarsal stair-step lateral extension of the transconjunctival approach demonstrated favorable early postoperative esthetic observations aided by its position within the natural crow’s-feet crease which is an advantage of this incision [16, 17, 21, 23] .

Our results were in accordance with those reported by Shannon R Garvey et al., who achieved favorable early cosmetic outcomes using the trans-tarsal modification of the transconjunctival approach, as the small cutaneous scar is effectively concealed within a natural skin crease [21]. So, in combination of the transconjunctival approach with the trans-tarsal extension within crow’s feet crease gives less scaring which gives it great advantage [23]. Similarly, Santosh and Giraddi reported that the transconjunctival approach offers favorable esthetic access to the infraorbital rim, orbital floor [1, 32]. During follow-up, transient entropion was observed in one case in each group and was managed appropriately in collaboration with the ophthalmology department. No persistent major eyelid malposition was encountered.

After the first postoperative day, pain levels were significantly higher in group B compared with group A. Both groups, however, exhibited a statistically significant reduction in pain at three weeks compared to the first week. These findings are consistent with the observations of Dickinson and Gausas, as well as the study by Shoukath et al., which attributed the decrease in pain to the corresponding reduction in postoperative edema [35, 36].

Regarding the sensory nerve function, there was statistically significant difference between the 2 groups at first, second, third weeks and up to 12 weeks postoperatively as group A had sensory nerve recovery at the malar area faster than group B. This was due to long term excessive edema caused by the soft tissue retraction and long procedure time in gaining reduction and fixation in group B. As any the surgical intervention induces inflammatory edema, which may exert pressure on the infraorbital nerve and subsequently lead to postoperative paraesthesia [37]. At the end of our follow-up period, which was 12 weeks, there was no statistically significant difference between the 2 groups.

It should be noted that severe comminuted inferior orbital rim fractures were excluded from this study. In cases with fragmented or severely displaced fractures, achieving adequate exposure using the transconjunctival approach alone may be more challenging, which could potentially influence operative manipulation, postoperative edema, and sensory outcomes. Therefore, the results of the present study regarding edema and sensory recovery may not be generalizable to such cases.

Radiographic evaluation was used as a confirmatory tool to verify proper fracture reduction and fixation, consistent with previously reported imaging-based postoperative assessment protocols [38].

The absence of a statistically significant difference in the primary outcome (exposure time) may reflect limited statistical power, given the sample size, to detect smaller, yet potentially clinically relevant, differences. Although normality was assessed using the Shapiro–Wilk test, the small sample size may limit the reliability of this assessment.

The relatively small sample size may limit the generalizability of the findings and increase the risk of type II error; however, the cohort size is comparable to previously published prospective OMC studies. Although more advanced statistical methods such as mixed-effects models could be considered, their application was limited by the sample size. Retrospective trial registration is also acknowledged as a limitation. Preoperative lower eyelid laxity was not specifically documented, although routine ophthalmologic evaluation was performed and cases with significant eyelid abnormalities were excluded. Eyelid malposition-related complications were not systematically recorded as predefined outcomes but were clinically monitored during follow-up. Several postoperative outcomes, including edema, pain, and scar appearance, were assessed subjectively by a single experienced examiner to reduce inter-observer variability; however, the lack of standardized assessment tools may affect objectivity and reproducibility. As well as complete assessor blinding was not always feasible during postoperative clinical evaluation. Finally, the 12-week follow-up period may not fully capture long-term outcomes, particularly scar maturation.

## Conclusion

Within the limitations of the current study, it can be concluded that the trans-tarsal stair-step lateral extension of the transconjunctival approach provides favorable clinical outcomes when compared to the conventional technique combined with a lateral eyebrow incision. The stair-step incision enables cosmetically advantageous access to the frontozygomatic region, resulting in early non-noticeable scar while eliminating the need for an additional lateral eyebrow incision. It also preserves the lateral canthal apparatus and avoids lateral canthotomy along with its associated complications. Moreover, suturing of the tarsal plate and eyelid margin are facilitated due to the clear identification of the gray line, which serves as a reliable anatomical landmark. Additionally, this approach offers wide exposure of the periorbital region without increasing the time required for surgical exposure. The trans-tarsal approach may represent a useful alternative for selected OMC fractures where continuous exposure and intraoperative accessibility are desired.

## Supplementary Information

Supplementary Material 1: Fig S1. Representative 3D CT image of the study group following the trans-tarsal stair-step incision, demonstrating proper reduction of the fracture and placement of fixation plates visually.

Supplementary Material 2.

## Data Availability

All data generated or analysed during this study are included in this published article.

## Abbreviations

OMC: Orbito-zygomaticomaxillary complex fractures
Trans-Tarsal: Trans-tarsal stair-step lateral extension of the transconjunctival approach
SNOSE: Sequentially Numbered, Opaque, Sealed Envelopes
NRS: Numerical rating scale
CT: Computed tomography
3D: 3-Dimensional

## References

[1] Melek LN, Noureldin MG. Zygomaticomaxillary complex fractures: finding the least complicated surgical approach (a randomized clinical trial). BMC Oral Health. 2023;23(1):539. 37542217 10.1186/s12903-023-03249-8 PMC10403894

[2] Melek LN, Sharara AA. Retrospective study of maxillofacial trauma in Alexandria University: Analysis of 177 cases. Tanta Dent J. 2016;13(1):28–33.

[3] Kurita M, Okazaki M, Ozaki M, Tanaka Y, Tsuji N, Takushima A, Harii K. Patient satisfaction after open reduction and internal fixation of zygomatic bone fractures. J Craniofac Surg. 2010;21(1):45–9. 20061977 10.1097/SCS.0b013e3181c36304

[4] Ji SY, Kim SS, Kim MH, Yang WS. Surgical methods of zygomaticomaxillary complex fracture. Archives Craniofac Surg. 2016;17(4):206. 10.7181/acfs.2016.17.4.206 PMC5556838 28913285

[5] Subramanian B, Krishnamurthy S, Suresh Kumar P, Saravanan B, Padhmanabhan M. Comparison of various approaches for exposure of infraorbital rim fractures of zygoma. J Oral Maxillofac Surg. 2009;8:99–102. 10.1007/s12663-009-0026-7 PMC3453937 23139484

[6] Waheed El-Anwar M, Elsheikh E, Sweed AH, Ezzeldin N. Electromyography assessment in zygomaticomaxillary complex fractures. Oral maxillofacial Surg. 2015;19:375–9. 10.1007/s10006-015-0505-6 25934247

[7] Kumar R, Ali R, Zaidi SAA, Maheshwari B, Ahmed K. Subciliary and Subtarsal incision in management of zygomatico-orbital fracture, a study on scar assessment. Pakistan J Med Health Sci. 2022;16(05):624–624.

[8] Novelli G, Ferrari L, Sozzi D, Mazzoleni F, Bozzetti A. Transconjunctival approach in orbital traumatology: a review of 56 cases. J Cranio-Maxillofacial Surg. 2011;39(4):266–70. 10.1016/j.jcms.2010.06.003 20650644

[9] El-Anwar MW, Elsheikh E, Hussein AM, Tantawy AA, Abdelbaki YM. Transconjunctival versus subciliary approach to the infraorbital margin for open reduction of zygomaticomaxillary complex fractures: a randomized feasibility study. Oral maxillofacial Surg. 2017;21(2):187–92. 10.1007/s10006-017-0617-2 28316023

[10] Wray RC Jr, Holtmann B, Ribaudo JM, Keiter J, Weeks PM. A comparison of conjunctival and subciliary incisions for orbital fractures. Br J Plast Surg. 1977;30(2):142–5. 858000 10.1016/0007-1226(77)90009-1

[11] Holtmann B, Wray RC, Little AG. A randomized comparison of four incisions for orbital fractures. Plast Reconstr Surg. 1981;67(6):731–5. 7243973 10.1097/00006534-198106000-00003

[12] McCord C Jr, Moses J. Exposure of the inferior orbit with fornix incision and lateral canthotomy. Ophthalmic Surg. 1979;10(6):53–63. 582852

[13] de Chalain TM, Cohen SR, Burstein FD. Modification of the transconjunctival lower lid approach to the orbital floor: lateral paracanthal incision. Plast Reconstr Surg. 1994;94(6):877–80. 7972439 10.1097/00006534-199411000-00023

[14] Biesman BS. Lateral paracanthal incision. Plast Reconstr Surg. 1995;96(7):1751. 7480311 10.1097/00006534-199512000-00056

[15] Jeong EC, Han J. Reply: Modified Transconjunctival Lower Lid Approach for Orbital Fractures in East Asian Patients: The Lateral Paracanthal Incision Revisited. Plast Reconstr Surg. 2015;136(1):118e. 25844521 10.1097/PRS.0000000000001340

[16] Kim D-W, Choi S-R, Park S-H, Koo S-H. Versatile use of extended transconjunctival approach for orbital reconstruction. Ann Plast Surg. 2009;62(4):374–80. 19325340 10.1097/SAP.0b013e3181855d27

[17] Song J, Lee GK, Kwon ST, Kim SW, Jeong EC. Modified transconjunctival lower lid approach for orbital fractures in East Asian patients: the lateral paracanthal incision revisited. Plast Reconstr Surg. 2014;134(5):1023–30. 25347636 10.1097/PRS.0000000000000639

[18] Yassin AM, Shaaban AM, Noureldin MG. Evaluation of the Y-shaped modification of the transconjuctival approach in open reduction of zygomatic maxillary complex fracture (clinical trial). Alexandria Dent J. 2022;47(1):9–15.

[19] Faul F, Erdfelder E, Buchner A, Lang A-G. Statistical power analyses using G* Power 3.1: Tests for correlation and regression analyses. Behav Res Methods. 2009;41(4):1149–60. 19897823 10.3758/BRM.41.4.1149

[20] Chung J-H, You H-J, Hwang N-H, Kim D-W, Yoon E-S. Transconjuctival incision with lateral paracanthal extension for corrective osteotomy of malunioned zygoma. Archives Craniofac Surg. 2016;17(3):119. 10.7181/acfs.2016.17.3.119 PMC5556799 28913268

[21] Garvey SR, Chen A, Nassar AH, Cauley RP. Trans-tarsal stair-step technique for lateral extension of the transconjunctival incision: a technical note and case series. Eplasty. 2024;24:e22. 38846500 PMC11155373

[22] Salgarelli AC, Bellini P, Landini B, Multinu A, Consolo U. A comparative study of different approaches in the treatment of orbital trauma: an experience based on 274 cases. Oral maxillofacial Surg. 2010;14(1):23–7. 10.1007/s10006-009-0176-2 19809838

[23] Olarte HF, Abello S, Castro-Núñez J. A modified lateral canthal approach for the treatment of zygomatic complex fractures. J Oral Maxillofac Surg. 2014;72(8):1552. e1551-1552. e1553. 10.1016/j.joms.2014.03.033 25037188

[24] Thangavelu K, Ganesh NS, Kumar JA, Sabitha S. Evaluation of the lateral orbital approach in management of zygomatic bone fractures. J Nat Sci Biology Med. 2013;4(1):117. 10.4103/0976-9668.107271 PMC3633260 23633846

[25] Uemura T, Chuman T, Fujii T, Morikawa A, Kikuchi M, Watanabe H. Retroseptal transconjunctival approach for blowout fracture of the orbital floor: an ideal choice in East-Asian patients. Plast Reconstr Surgery–Global Open. 2016;4(5):e725. 10.1097/GOX.0000000000000683 PMC4995712 27579249

[26] Kumar S, Shubhalaksmi S. Clinical outcome following use of transconjunctival approach in reducing orbitozygomaticomaxillary complex fractures. Contemp Clin Dent. 2016;7(2):163–9. 27307661 10.4103/0976-237X.183067 PMC4906857

[27] Kumar P, Godhi S, Lall AB, Ram C. Evaluation of neurosensory changes in the infraorbital nerve following zygomatic fractures. J Oral Maxillofac Surg. 2012;11(4):394–9. 10.1007/s12663-012-0348-8 PMC3485474 24293929

[28] Johnson C. Measuring pain. Visual analog scale versus numeric pain scale: what is the difference? J Chiropr Med. 2005;4(1):43. 19674646 10.1016/S0899-3467(07)60112-8 PMC2647033

[29] Kesselring AG, Promes P, Strabbing EM, van der Wal KG, Koudstaal MJ. Lower eyelid malposition following orbital fracture surgery: a retrospective analysis based on 198 surgeries. Craniomaxillofacial trauma reconstruction. 2016;9(2):109–12. 27162565 10.1055/s-0035-1567813 PMC4858425

[30] Soliman L, Rhee B, Lerner JL, Sobti N, Rao V, Woo AS. Lateral canthotomy revisited: a refined surgical approach for orbital access. Plast Reconstr Surgery–Global Open. 2023;11(5):e5014. 10.1097/GOX.0000000000005014 PMC10287141 37360241

[31] Bonawitz S, Crawley W, Shores JT, Manson PN. Modified transconjunctival approach to the lower eyelid: technical details for predictable results. Craniomaxillofacial Trauma Reconstruction. 2016;9(1):029–34. 10.1055/s-0035-1556051 PMC4755724 26889345

[32] Santosh B, Giraddi G. Transconjunctival preseptal approach for orbital floor and infraorbital rim fracture. J Oral Maxillofac Surg. 2011;10(4):301–5. 10.1007/s12663-011-0246-5 PMC3267922 23204744

[33] da Silveira JSZ. Edema Management in Oral and Maxillofacial Surgery. Inflammation in the 21st Century 2022:59. 10.5772/intechopen.80971.

[34] Kashiyama K, Yano H, Imamura Y, Iwao A, Higashi A, Moriuti Y, Ashizuka S, Adachi Y, Koga K, Hirano A. Scarring Caused by the Percutaneous Approach to Fractures of the Orbit and Orbital Rim. J Craniofac Surg. 2022;33(4):1143–6. 34739449 10.1097/SCS.0000000000008312

[35] Shoukath S, Taylor GI, Mendelson BC, Corlett RJ, Shayan R, Tourani SS, Ashton MW. The lymphatic anatomy of the lower eyelid and conjunctiva and correlation with postoperative chemosis and edema. Plast Reconstr Surg. 2017;139(3):e628–37. 10.1097/PRS.0000000000003094 28234825

[36] Dickinson A, Gausas R. Orbital lymphatics: do they exist? Eye. 2006;20(10):1145–8. 17019412 10.1038/sj.eye.6702378

[37] Lakshmi R, Chitra A, Singh A, Pentapati KC, Gadicherla S. Neurosensory assessment of infraorbital nerve injury following unilateral zygomaticomaxillary complex fracture–a prospective study. Open Dentistry J. 2022;16(1). 10.2174/18742106-v16-e2206140.

[38] de Carvalho M, Vieira J, Figueiredo R, Reher P, Chrcanovic BR, Chaves M. Validity of computed tomography in diagnosing midfacial fractures. Int J Oral Maxillofac Surg. 2021;50(4):471–6. 32980217 10.1016/j.ijom.2020.09.002

