# Supplementary figures and images for: Trans-tarsal stair-step versus transconjunctival approach in orbito-zygomaticomaxillary fractures: a prospective randomized controlled clinical trial
Source: BMC Oral Health. 2026 Jun 17;26:1069. doi: 10.1186/s12903-026-08842-1 (PMC13274052; doi:10.1186/s12903-026-08842-1)

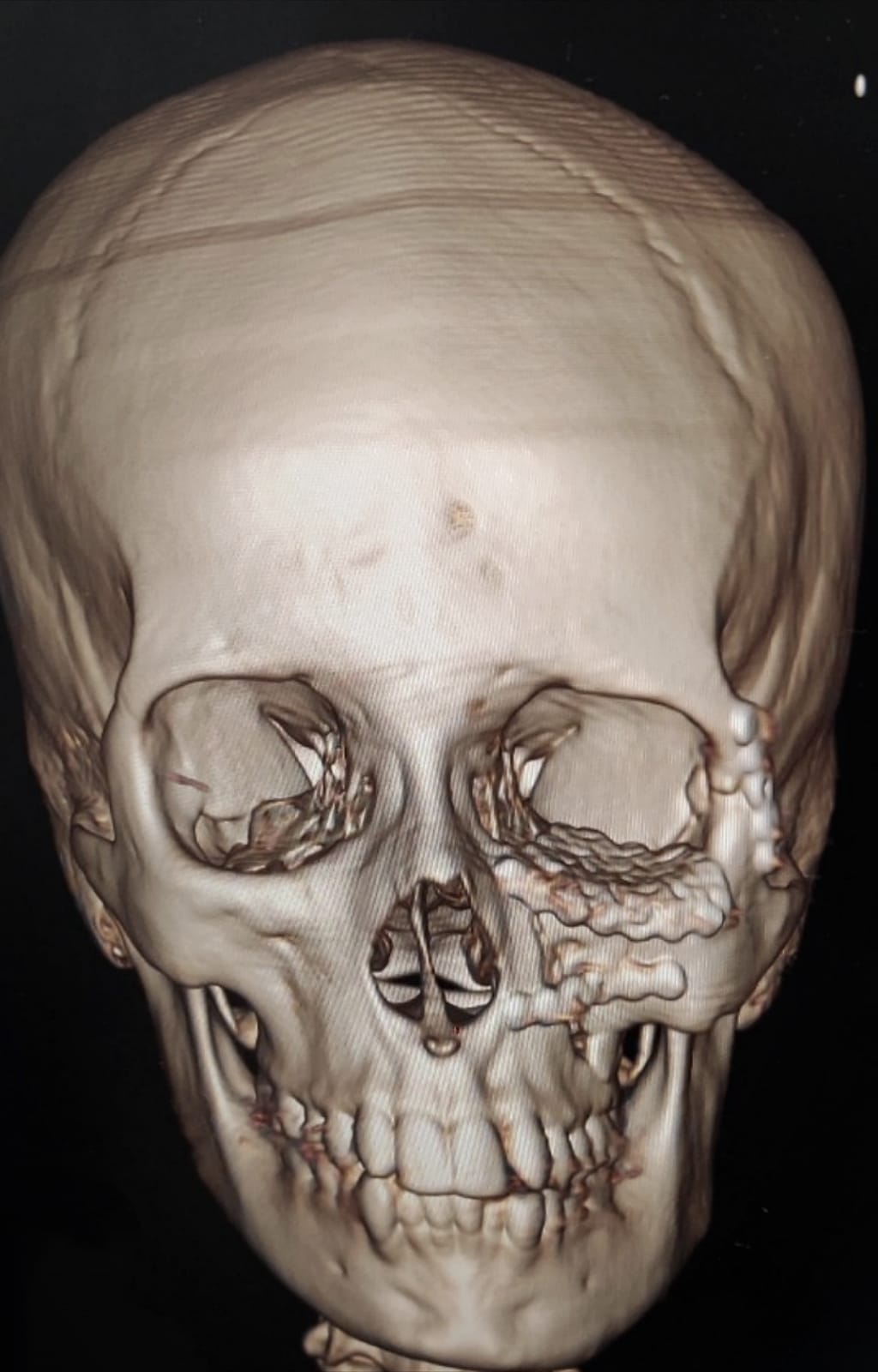

Supplement: Supplementary file 1 — Supplementary Material 1: Fig S1. Representative 3D CT image of the study group following the trans-tarsal stair-step incision, demonstrating proper reduction of the fracture and placement of fixation plates visually. [file 12903_2026_8842_MOESM1_ESM.jpg]
